# Radiotherapy for Metastatic Epidural Spinal Cord Compression with Increased Doses: Final Results of the RAMSES-01 Trial

**DOI:** 10.3390/cancers16061149

**Published:** 2024-03-14

**Authors:** Dirk Rades, Darejan Lomidze, Natalia Jankarashvili, Fernando Lopez Campos, Arturo Navarro-Martin, Barbara Segedin, Blaz Groselj, Christian Staackmann, Charlotte Kristiansen, Kristopher Dennis, Steven E. Schild, Jon Cacicedo

**Affiliations:** 1 Department of Radiation Oncology, University of Lubeck, 23562 Lubeck, Germany; christian.staackmann@uksh.de; 2 Radiation Oncology Department, Tbilisi State Medical University, 0186 Tbilisi, Georgia; dlomidze@hotmail.com; 3 Ingorokva High Medical Technology University Clinic, 0144 Tbilisi, Georgia; 4 Department of Radiation Oncology, Acad. F. Todua Medical Center—Research Institute of Clinical Medicine, 0112 Tbilisi, Georgia; natnataliaj@yahoo.com; 5 Department of Radiation Oncology, University Hospital Ramón y Cajal, 28034 Madrid, Spain; flcampos@salud.madrid.org; 6 Department of Radiation Oncology, Instituto Catalán de Oncología, 08908 l’Hospitalet de Llobregat, Spain; anavarro@iconcologia.net; 7 Department of Radiotherapy, Institute of Oncology Ljubljana and Faculty of Medicine, University of Ljubljana, 1000 Ljubljana, Slovenia; bsegedin@onko-i.si (B.S.); bgroselj@onko-i.si (B.G.); 8 Department of Oncology, Vejle Hospital, University Hospital of Southern Denmark, 7100 Vejle, Denmark; charlotte.kristiansen@rsyd.dk; 9 Division of Radiation Oncology, The Ottawa Hospital and the University of Ottawa, Ottawa, ON K1Y 4E9, Canada; 10 Department of Radiation Oncology, Mayo Clinic, Phoenix, AZ 85054, USA; sschild@mayo.edu; 11 Department of Radiation Oncology, Cruces University Hospital/Biobizkaia Health Research Institute, 48903 Barakaldo, Spain; jon.cacicedofernandezbobadilla@osakidetza.eus

**Keywords:** metastatic epidural spinal cord compression, highly conformal radiotherapy, increased doses, phase 2 trial, historical control group, local progression-free survival

## Abstract

**Simple Summary:**

Patients with MESCC and favorable survival prognoses assigned to radiotherapy alone may benefit from increased doses. In a multi-center phase 2 trial, patients receiving 15 × 2.633 Gy or 18 × 2.333 Gy were evaluated and subsequently compared to a historical control group receiving 10 × 3.0 Gy. The phase 2 cohort, including 50 (of 62 planned) evaluable patients, showed promising results regarding 12-month local progression-free survival (LPFS), 12-month overall survival (OS), improvement of motor and sensory functions, post-radiotherapy ambulatory status, and relief of pain and distress. Radiotherapy with 15 × 2.633 Gy or 18 × 2.333 Gy was well tolerated and appeared more effective than 10 × 3.0 Gy with respect to LPFS and improvement of motor function.

**Abstract:**

Patients with metastatic epidural spinal cord compression (MESCC) and favorable survival prognoses may benefit from radiation doses exceeding 10 × 3.0 Gy. In a multi-center phase 2 trial, patients receiving 15 × 2.633 Gy (41.6 Gy_10_) or 18 × 2.333 Gy (43.2 Gy_10_) were evaluated for local progression-free survival (LPFS), motor/sensory functions, ambulatory status, pain, distress, toxicity, and overall survival (OS). They were compared (propensity score-adjusted Cox regression) to a historical control group (n = 266) receiving 10 × 3.0 Gy (32.5 Gy_10_). In the phase 2 cohort, 50 (of 62 planned) patients were evaluated for LPFS. Twelve-month rates of LPFS and OS were 96.8% and 69.9%, respectively. Motor and sensory functions improved in 56% and 57.1% of patients, and 94.0% were ambulatory following radiotherapy. Pain and distress decreased in 84.4% and 78.0% of patients. Ten and two patients experienced grade 2 and 3 toxicities, respectively. Phase 2 patients showed significantly better LPFS than the control group (*p* = 0.039) and a trend for improved motor function (*p* = 0.057). Ambulatory and OS rates were not significantly different. Radiotherapy with 15 × 2.633 Gy or 18 × 2.333 Gy was well tolerated and appeared superior to 10 × 3.0 Gy.

## 1. Introduction

Spinal metastases are relatively common among patients with metastatic disease, particularly in those with lung cancer, breast cancer, prostate cancer, myeloma, or renal cell carcinoma [1]. For many of these patients, spinal metastases are associated with metastatic epidural spinal cord compression (MESCC) and consequently motor and sensory deficits. Select patients with a good performance status, an estimated survival time of at least 3 months, the involvement of only one spinal segment by MESCC, and not overly radiosensitive tumors were demonstrated to benefit from the addition of upfront decompressive surgery to radiotherapy [2]. However, many patients with MESCC receive radiotherapy alone. For these patients, several dose fractionation regimens are available [1]. These include single-fraction, multi-fraction short-course (overall treatment time approximately one week), and multi-fraction long-course (overall treatment time 2–4 weeks) programs. It is generally agreed that patients with poor to intermediate survival prognoses should be treated with short-course or, in case of very poor prognoses, single-fraction radiotherapy [1,3,4,5].

However, many patients irradiated for MESCC have more favorable survival prognoses. These patients were shown to benefit from longer-course radiotherapy, particularly in terms of better local control (LC) and local progression-free survival (LPFS) of MESCC, when compared to shorter programs [6]. The most common longer-course program used for MESCC worldwide is 10 × 3.0 Gy over 2 weeks [1]. In a retrospective matched-pair study of 382 patients with MESCC and favorable survival prognoses, longer-course radiotherapy with higher doses, namely 15 × 2.5 Gy [equivalent dose in 2 Gy fractions (EQD2) = 39.1 Gy_10_] and 20 × 2 Gy (EQD2 = 40.0 Gy_10_), resulted in significantly better long-term LC, LPFS, and overall survival (OS) when compared to 10 × 3.0 Gy (EQD2 = 32.5 Gy_10_) [7,8,9]. These findings led to the current RAMSES-01 trial, which investigated whether further incremental dose escalation could improve outcomes. Patients included in this phase 2 trial had favorable survival prognoses and were scheduled for highly conformal radiotherapy with 18 × 2.333 Gy (EQD2 = 43.2 Gy_10_) or 15 × 2.633 Gy (EQD2 = 41.6 Gy_10_). In addition, patients of the RAMSES-01 cohort were compared to a historical control group treated with 10 × 3.0 Gy of conventional radiotherapy. The major goal of this study was to show that highly conformal radiotherapy with increased doses results in better LPFS when compared to the commonly used regimen 10 × 3.0 Gy.

## 2. Materials and Methods

In this multi-center phase 2 trial (RAMSES-01), the outcomes of highly conformal radiotherapy with increased doses were investigated in patients with motor deficits due to MESCC and favorable survival prognoses assigned to radiotherapy alone without upfront surgery [10]. Highly conformal radiotherapy included volumetric modulated arc therapy (VMAT) and intensity-modulated radiation therapy (IMRT). A favorable prognosis was defined as ≥36 points on a validated survival score that incorporates six independent prognostic factors, including tumor type, additional osseous metastases, metastatic spread to other organs, the time period between initial diagnosis of the malignant disease and occurrence of MESCC, gait function prior to irradiation of MESCC, and number of days during which motor deficits developed [11]. This study was approved by responsible ethics committees (leading committee: University of Lübeck, 18-360), performed in accordance with the Declaration of Helsinki, and registered at clinicaltrials.gov (NCT04043156).

The initial dose fractionation regimen of the RAMSES-01 trial was 18 × 2.333 Gy, representing an EQD2 of 43.2 Gy_10_ for tumor cell kill (α/β 10 Gy). This represented an increase in the EQD2 by 33% compared to 10 × 3.0 Gy (EQD2 = 32.5 Gy_10_) [8,9]. When the COVID-19 pandemic started, the question arose of whether it was reasonably possible to reduce the number of radiotherapy sessions. It was decided to offer 15 × 2.633 Gy as an alternative, which represents an EQD2 of 41.6 Gy_10_ and an increase in the EQD2 by 28% compared to 10 × 3.0 Gy [7,8]. The maximum relative doses allowed to the spinal cord were 101.5% of the prescribed dose for 18 × 2.333 Gy and 101.2% for 15 × 2.633 Gy. Both doses represented an EQD2 of 46.6 Gy_2_ for myelopathy (α/β 2 Gy) [7,8].

Patients included must have had motor deficits of at least one leg for a maximum duration of 30 days. Compression of the spinal cord was confirmed by magnetic resonance imaging (preferred) or computed tomography. Prior to radiotherapy, patients were presented to a surgeon to assess the necessity for decompressive surgery. Those operated on were ineligible. During the radiotherapy course, patients were recommended to receive concomitant treatment with dexamethasone. The primary endpoint of the RAMSES-01 trial was LPFS at 12 months following radiotherapy. LPFS was defined as no deterioration of motor function during and no in-field recurrence of MESCC following radiotherapy. Secondary endpoints included improvement of motor and sensory functions, gait function following irradiation, relief, decrease in distress, adverse events, and OS. Motor function was assessed using a 5-point scale (0 = strength not impaired, 1 = patient can walk without aid, 2 = patient can walk but aid (e.g., walker or crutches) required, 3 = patient is unable to walk, 4 = patient is suffering from complete paraplegia), sensory function impaired vs. normal [12,13]. Improvement and deterioration of motor deficits were defined as a change in motor function by ≥1 point [12]. The intensity of pain was rated with a numeric self-assessment scale, ranging between 0 points (no pain) and 10 points (maximum pain) [14]. Partial pain relief was defined as an improvement by ≥2 points from the baseline without an increase in daily opioid analgesics or a reduction in opioid analgesics by at least 25% without an increase in pain [14]. Complete pain relief was defined as a decrease in pain to 0. Distress was evaluated using the distress thermometer, also ranging between 0 points (no distress) and 10 points (maximum distress) [15]. Relief of distress was defined as a decrease of ≥2 points from the baseline. The Common Terminology Criteria for Adverse Events (CTCAE) version 4.03 was used to grade toxicities [16]. These endpoints were assessed directly and at 1, 3, 6, 9, and 12 months following radiotherapy. In the case of an out-field recurrence of MESCC, patients were censored for LPFS. Further details of the study protocol were reported previously [10].

Patients of the RAMSES-01 trial were compared to a historical control group from multiple institutions with motor deficits due to MESCC and favorable survival prognoses who were irradiated with 10 × 3.0 Gy of conventional radiotherapy, using propensity score-adjusted analyses. The baseline characteristics of both treatment groups are summarized in Table 1. Both groups were compared with respect to LPFS, OS, improvement of motor function, and post-radiotherapy ambulatory status. In order to avoid bias due to different lengths of follow-up, the follow-up period in the historical control group was limited to 12 months.

### Statistical Considerations

For the RAMSES-01 trial, a required sample size of *n* = 62 was calculated [10]. Assuming that 5% of the enrolled patients would not be eligible, recruitment of 65 patients was planned. The evaluation was performed in patients who were available for assessment of the primary endpoint and received at least 80% of the planned radiation dose, i.e., 15 of 18 fractions with 2.333 Gy and 12 of 15 fractions with 2.633 Gy, respectively. LPFS and OS rates were calculated with the Kaplan–Meier method. For the comparison of the RAMSES-01 cohort and the historical control group, the propensities were estimated using several baseline characteristics that are shown in Table 1 [10]. To obtain valid maximum likelihood estimates, time-developing motor deficits were aggregated and included in the respective logistic regression model as a binary variable with values ≤ 14 days and >14 days. The Hosmer–Lemeshow Goodness-of-Fit Test was *p* = 0.54.

After the propensity scores were estimated, several methods and their variations existed to create a balance between the RAMSES-01 cohort and the prospective and historical control group. However, due to the low number of expected events, commonly used approaches, such as one-to-one matching, stratification, and inverse probability of treatment weighting, were not recommended. Instead, regression on the propensity score by estimating a Cox proportional hazards model for the outcome of interest with independent variables for treatment and the logit of the propensity scores was applied. The regression model was chosen to allow for a non-linear association between the propensity score and the outcome link function by means of the ‘one-spline’ approach, as described by Franklin et al. [17]. To reduce any potential bias, this modeling approach was already pre-specified in the study protocol. In particular, a restricted cubic spline transformation was used, consisting of cubic functions between the knots and linear functions in the tails, which allowed for many possible complex forms. Five knots were placed at equally spaced percentiles of the log odds. To compare the treatment groups with respect to their effect on the improvement of motor function (yes vs. no) and post-radiotherapy ambulatory status (yes vs. no), a logistic regression model was applied. For adjustment, the same propensities as above were used. The statistical analyses were performed with SAS software (version 9.4; SAS, Cary, NC, USA).

## 3. Results

### 3.1. RAMSES-01 Trial

For the RAMSES-01 trial, 52 of the 65 planned patients were recruited between 08/2019 and 11/2021. Since during this study OS was found to be worse than expected, a new survival score was developed, which was more precise in predicting OS than the tool used for the RAMSES-01 trial [18]. As a consequence, the RAMSES-01 trial was terminated. Two of the fifty-two enrolled patients died during the radiotherapy course after six of eighteen fractions (sepsis) and twelve of eighteen fractions (acute decompensated heart failure), respectively, and were not evaluable for LPFS. In accordance with the study protocol, these patients were not included in the analyses. Of the remaining fifty patients, forty-one patients (82%) received VMAT and nine patients (18%) IMRT, respectively. Of the thirteen patients treated with eighteen fractions, twelve patients received 18 × 2.333 Gy and one patient treated with concurrent immunotherapy received 15 × 2.333 + 3 × 2.0 Gy (EQD2 = 42.0 Gy_10_, an increase of 29% over 10 × 3.0 Gy). Of the thirty-seven patients treated with fifteen fractions, thirty-three patients received 15 × 2.633 Gy, three patients treated with concurrent immunotherapy received 12 × 2.633 + 3 × 2.333 Gy (EQD2 = 40.5 Gy_10_, an increase of 25%), and one emergency patient who started with 3.0 Gy received 1 × 3.0 Gy + 12 × 2.633 + 2 × 2.333 (EQD2 = 41.4 Gy_10_, an increase of 27%), respectively. Twenty-eight patients received concurrent dexamethasone (median 8 mg/day, range: 4–16 mg/day). The other patients had contraindications, refused to take corticosteroids, or had very mild motor impairment. Since all 50 patients received at least 80% of the planned dose per protocol, they were eligible for the analyses.

In these 50 patients, 12-month rates of LPFS and OS were 96.8% [95% CI: (79.2%; 99.5%)] and 69.9% [95% CI: (55.1%; 80.6%)], respectively. LPFS rates at 3, 6, and 9 months following radiotherapy were 100%, 100%, and 96.8%, respectively, and OS rates were 88%, 74%, and 72%, respectively. During the first 6 months following radiotherapy, improvement of motor function occurred in 28 patients (56.0%). Two additional patients (4.0%) improved after 9 months. Forty-seven patients (94.0%) were ambulatory following radiotherapy, including forty-five patients who were ambulatory prior to radiotherapy and two non-ambulatory patients. Of the twenty-seven patients who were ambulatory with aid prior to radiotherapy, eight patients (29.6%) regained normal strength and another eleven patients (40.7%) became ambulatory without aid.

Within 6 months following radiotherapy, 12 of the 21 patients (57.2%) with pre-radiotherapy sensory deficits improved. One additional patient (4.8%) improved after 9 months. Thirty-eight of the forty-five patients with pre-radiotherapy pain (84.4%) experienced at least partial relief (best response) within 6 months following radiotherapy, of whom ten patients (22.2%) achieved complete relief. Forty-one patients (82.0%) reported relief of distress within 6 months following radiotherapy. Grade 2 toxicities (mainly esophagitis/dysphagia) occurred in ten patients (20.0%) and grade 3 toxicities in another two patients (one diarrhea, one esophagitis). Late sequelae, such as myelopathy or vertebral fractures, did not occur.

### 3.2. Comparison to the Historical Control Group

**Figure 1:**
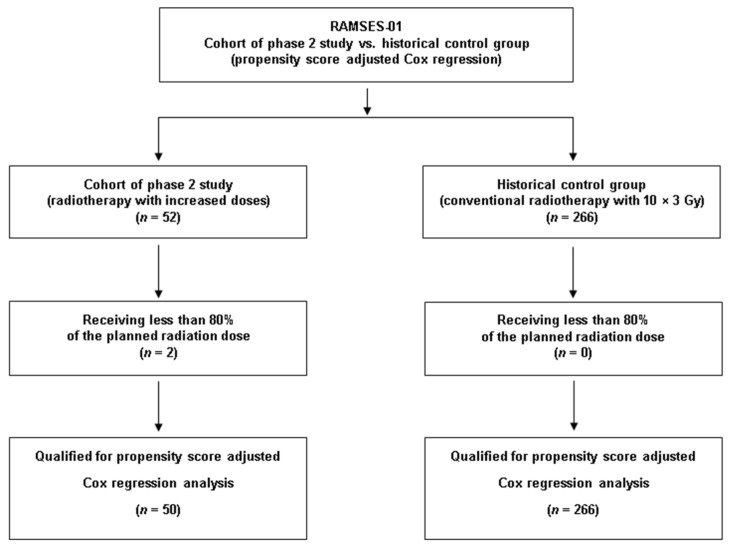
CONSORT diagram for comparison of the RAMSES-01 cohort and the historical control group.

**Figure 2:**
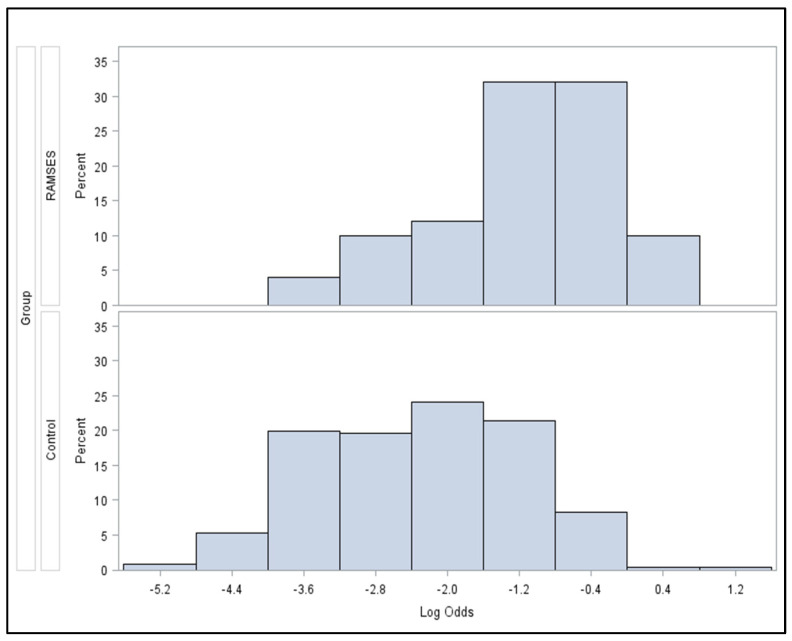
Distribution of the propensity scores stratified by patient cohort on the logit scale (*p* < 0.001, Wilcoxon two-sample test).

**Figure 3:**
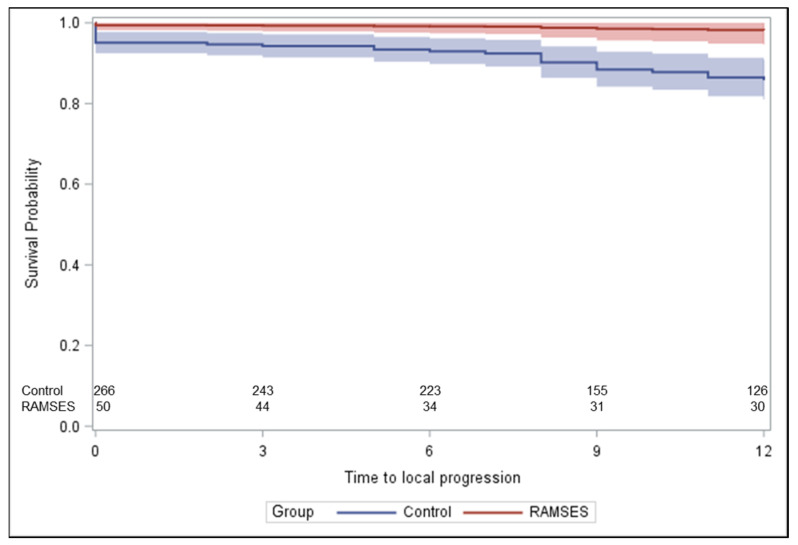
Estimated local progression-free survival (LPFS) after propensity score adjustment. The curves show the direct adjusted local-free progression curves together with their 95% confidence limits (shaded areas), defined as the average of the predicted LPFS curves for all group-specific patients based on the propensity score-adjusted proportional hazards model.

**Figure 4:**
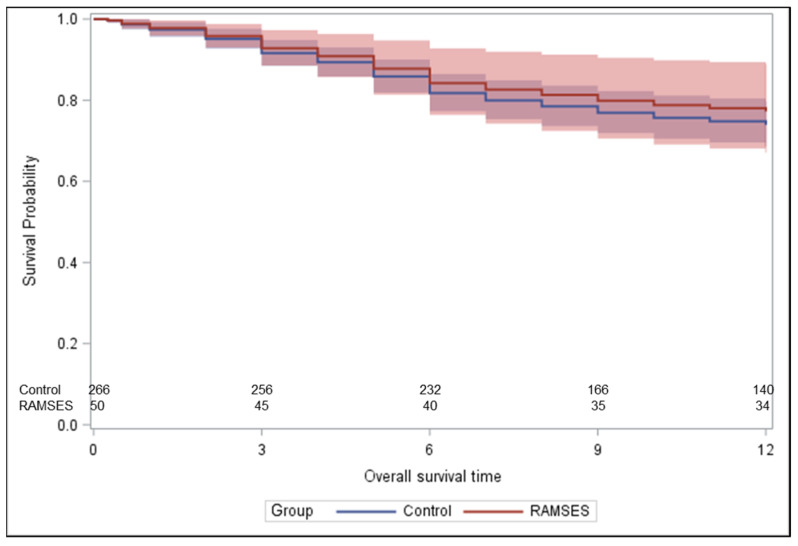
Estimated overall survival (OS) after propensity score adjustment. The curves show the direct adjusted overall survival curves together with their 95% confidence limits (shaded areas), defined as the average of the predicted overall survival curves for all group-specific patients based on the propensity score-adjusted proportional hazards model.

Two-hundred-sixty-six patients treated between 1992 and 2022 met the criteria for the control group, including a reception of ≥80% of the planned dose of 10 × 3.0 Gy (Figure 1). The corresponding baseline characteristics are shown in Table 1. These patients received concurrent dexamethasone with doses of 4–32 mg/day. In the majority of the patients included in the historical control group, radiotherapy was performed through a posterior field (dose prescribed to anterior vertebra) or parallel opposed fields (dose prescribed to the midplane). Figure 2 demonstrates the statistically significant heterogeneity of the propensity score distributions between the groups and the need for adjustment. After propensity score adjustment, LPFS was significantly better in patients in the RAMSES-01 trial when compared to the patients in the historic control group (hazard ratio 0.116, *p* = 0.039, Figure 3). Moreover, patients in the RAMSES-01 cohort showed a trend with respect to improvement of motor function (odds ratio 1.943, *p* = 0.057). Post-radiotherapy ambulatory rates (odds ratio 1.484, *p* = 0.56) and OS rates (hazard ratio 0.851, *p* = 0.62, Figure 4) were not significantly different. The results of the unadjusted/crude and propensity score-adjusted analyses regarding LPFS, improvement of motor function, post-radiotherapy ambulatory status, and OS are shown in Table 2.

## 4. Discussion

In 2011, a retrospective matched-pair study was performed in patients with favorable survival prognoses receiving conventional radiotherapy for motor deficits due to MESCC [9]. This study compared 191 patients receiving 10 × 3.0 Gy (EQD2 = 32.5 Gy_10_) to 191 patients treated with 15 × 2.5 Gy (EQD2 = 39.1 Gy_10_) or 20 × 2.0 Gy (EQD2 = 40.0 Gy_10_). The higher doses were associated with significantly improved LC of MESCC (92% vs. 87% at 12 months, *p* = 0.012), LPFS (90% vs. 84%, *p* = 0.013), and OS (81% vs. 76%, *p* = 0.032) [9]. Since highly conformal radiotherapy techniques, such as VMAT and IMRT, provide better sparing of normal tissues, including the spinal cord, and these techniques allow an increase in the EQD2 beyond 40.0 Gy without exceeding the tolerance dose of the spinal cord of 45–50 Gy [19,20]. In the RAMSES-01 trial, patients received VMAT or IMRT with an EQD2 of 40.5 Gy_10_ to 43.2 Gy_10_ for tumor cell kill (≥41.4 Gy_10_ in 94% of patients) and a maximum EQD2 of 46.6 Gy_2_ for myelopathy [7,8]. Patients in the RAMSES-01 cohort were compared to a historical control group treated with 10 × 3.0 Gy, the most common longer-course regimen for MESCC.

During the phase 2 trial, we realized that the survival of the patients was worse than expected. This finding may be explained by the fact that since the Patchell trial was published, many patients with longer estimated survival times receive upfront surgery, and those assigned to radiotherapy alone have comparably less favorable prognoses [2]. Therefore, we decided to develop a new survival score based on the data of patients treated with radiotherapy alone within prospective trials [18]. The new scoring tool was based on three prognostic factors, namely, the type of primary tumor, pre-radiotherapy ambulatory status, and visceral metastases. It was more accurate in identifying patients who live 6 months or longer when compared to the previous score [11,18]. Corresponding positive predictive values were 90% and 64%, respectively. Since the RAMSES-01 trial was based on the previous survival score, the decision was made to stop the recruitment. At that time, 52 patients (80% of the planned sample size) had already been enrolled, of whom 50 patients were evaluable for the primary endpoint LPFS and, therefore, eligible for the planned analyses.

The outcomes of these 50 patients were very promising with respect to LPFS (96.8%), improvement of motor function (56.0%), improvement of sensory function (57.2%), post-radiotherapy ambulatory status (94.0%), pain relief (84.4%), and relief of distress (78.0%). Moreover, the dose fractionation regimens used in the phase 2 part of our study were sufficiently well tolerated, with grade 3 toxicities occurring in only two patients (4%). Furthermore, late radiation-related toxicity was not observed during the period of follow-up. When compared to the results of the previous matched-pair study, the 12-month LPFS rate in the RAMSES-01 cohort was higher [9]. This applied to both patients receiving 10 × 3.0 Gy (96.8% vs. 84%) and patients receiving 15 × 2.5 Gy or 20 × 2 Gy (96.8% vs. 90%). In addition, improvement of motor function was more frequent in the RAMSES-01 cohort than in the treatment groups of the matched-pair study (56.0% vs. 41% and 40%, respectively). Moreover, the rate of pain relief was higher than the rates of 58–81% reported in previous prospective trials for patients irradiated for painful bone metastases [21,22,23,24,25]. The favorable outcomes of the dose fractionation regimens used in the RAMSES-01 trial were confirmed in the second part of the present study, the comparison to the historic control group treated with 10 × 3.0 Gy. The RAMSES-01 regimens resulted in significantly better LPFS and showed a strong trend for a higher rate of improvement of motor function.

### Limitations of This Study

Although dose fractionation regimens of the RAMSES-01 trial appear preferable for patients with MESCC and favorable survival prognoses, the limitations of this study should be considered. Its major limitation is the fact that this study had to be closed after the inclusion of 80% of the planned sample size. Another limitation of this study is the fact that four patients (8%) received an EQD2 of less than 41.6 Gy_10_, an EQD2 of 15 × 2.633 Gy. Also, the retrospective nature of the historical control group must be considered. Moreover, the majority of the patients in the control group did not receive highly conformal radiotherapy with VMAT or IMRT. Despite the procedure of propensity score-adjusted Cox regression, hidden selection biases cannot be entirely eliminated. One should also consider that two patients in the RAMSES-01 cohort, who died prior to completion of their radiotherapy course, were not included in the analyses. This may have led to an additional bias, although the patients died from causes other than MESCC or radiation-related toxicity. An additional limitation of this study is given by the fact that pathological changes and inflammatory markers were not investigated.

Selected patients with single or very few lesions may be candidates for other highly conformal techniques, such as stereotactic body radiation therapy (SBRT) [26,27,28]. Moreover, the results of this study may not apply to patients with MESCC from metastatic sarcoma or patients with MESCC and large soft tissue components or soft tissue metastases [29]. Due to its limitations, this study should be considered hypothesis generating and not practice changing. This holds true, although the main study objective, namely, demonstrating that the dose fractionation regimens used in the RAMSES-01 trial led to improved LPFS, has been achieved.

## 5. Conclusions

Highly conformal radiotherapy with 15 × 2.633 Gy or 18 × 2.333 Gy was sufficiently well tolerated and resulted in significantly better long-term LPFS than 10 × 3.0 Gy in patients with MESCC and favorable survival prognoses. Given the limitations of the present study, the dose fractionation regimens of the RAMSES-01 trial appear preferable for these patients if they are candidates for radiotherapy alone without upfront surgery. Additional prospective trials are required to define the optimal dose fractionation regimen of radiotherapy for MESCC in patients with favorable survival prognoses. For example, a further increase in the radiation dose should be investigated in future trials. In those trials, all patients should be presented to a neurosurgeon or orthopedic surgeon to evaluate the indication for upfront surgery considering spinal instability [2,30,31,32,33,34,35,36,37,38,39]. 

## Figures and Tables

**Table 1:** Baseline characteristics of the RAMSES-01 cohort (n = 50) and the historical control group treated with 10 × 3.0 Gy (n = 266) without propensity score adjustment.

Characteristic	RAMSES n Patients (%)	Control Group n Patients (%)
Age		
≤64 years	23 (46.0)	132 (49.6)
>65 years	27 (54.0)	134 (50.4)
Gender		
Female	18 (36.0)	137 (51.5)
Male	32 (64.0)	129 (48.5)
Interval tumor diagnosis of MESCC		
≤15 months	25 (50.0)	96 (36.1)
>15 months	25 (50.0)	170 (63.9)
Visceral metastases		
No	44 (88.0)	248 (93.2)
Yes	6 (12.0)	18 (6.8)
Other bone metastases		
No	9 (18.0)	121 (45.5)
Yes	41 (82.0)	145 (54.5)
Type of primary tumor		
Breast cancer	13 (26.0)	98 (36.8)
Prostate cancer	15 (30.0)	61 (22.9)
Myeloma/lymphoma	10 (20.0)	55 (20.7)
Lung cancer	4 (8.0)	10 (3.8)
Other tumors	8 (16.0)	43 (15.8)
Time-developing motor deficits		
1–7 days	0 (0.0)	22 (8.3)
8–14 days	8 (16.0)	74 (27.8)
>14 days	42 (84.0)	170 (63.9)
Pre-radiotherapy ambulatory status		
Not ambulatory	5 (10.0)	39 (14.7)
Ambulatory	45 (90.0)	227 (85.3)
Number of affected vertebrae		
1–2	30 (60.0)	145 (54.5)
≥3	20 (40.0)	121 (45.5)
ECOG performance score		
0–2	32 (64.0)	195 (73.3)
3–4	18 (36.0)	71 (26.7)

ECOG: Eastern Cooperative Oncology Group; MESCC: metastatic epidural spinal cord compression.

**Table 2:** Comparison of the RAMSES-01 cohort and the historical control group with respect to local progression-free survival and overall survival (using a Cox regression model and propensity score adjustment), and improvement of motor function, and post-radiotherapy ambulatory status (using a logistic regression model and propensity score adjustment).

Endpoint	Hazard Ratio Estimate	95% Confidence Interval	*p*-Value
Local progression-free survival			
Unadjusted/crude	0.181	0.025–1.329	0.093
Propensity score adjusted ^a^	0.116	0.015–0.894	0.039
Overall survival			
Unadjusted/crude	1.303	0.741–2.290	0.36
Propensity score adjusted ^b^	0.851	0.453–1.598	0.62
Improvement of motor function			
Unadjusted/crude	1.777	0.966–3.268	0.064
Propensity score adjusted ^c^	1.943	0.981–3.850	0.057
Post-radiotherapy ambulatory status			
Unadjusted/crude	1.483	0.428–5.140	0.54
Propensity score adjusted ^d^	1.464	0.373–5.738	0.58

^a–d^ With adjustment for the knots for the spline effect of the logit-transformed propensity score. ^a^ Wald chi-square test with four degrees of freedom, *p* = 0.004. ^b^ Wald chi-square test with four degrees of freedom, *p* = 0.004. ^c^ Wald chi-square test with four degrees of freedom, *p* = 0.075. ^d^ Wald chi-square test with four degrees of freedom, *p* = 0.48. Bold *p*-values are significant.

## Data Availability

Data from the RAMSES-01 trial are available at clinicaltrials.gov (identifier NCT04043156). Otherwise, the data analyzed in this paper cannot be shared due to data protection regulations.

## References

[1] Prasad D., Schiff D. (2005). Malignant spinal cord compression. Lancet Oncol..

[2] Patchell R., Tibbs P.A., Regine W.F., Payne R., Saris S., Kryscio R.J., Mohiuddin M., Young B. (2005). Direct decompressive surgical resection in the treatment of spinal cord compression caused by metastatic cancer: A randomised trial. Lancet.

[3] Hoskin P.J., Hopkins K., Misra V., Holt T., McMenemin R., Dubois D., McKinna F., Foran B., Madhavan K., MacGregor C. (2019). Effect of single-fraction vs multifraction radiotherapy on ambulatory status among patients with spinal canal compression from metastatic cancer: The SCORAD randomized clinical trial. JAMA.

[4] Thirion P.G., Dunne M.T., Kelly P.J., Flavin A., O’Sullivan J.M., Hacking D., Sasiadek W., Small C., Pomeroy M.M., Martin J. (2020). Non-inferiority randomised phase 3 trial comparing two radiation schedules (single vs. five fractions) in malignant spinal cord compression. Br. J. Cancer.

[5] Rades D., Šegedin B., Conde-Moreno A.J., Garcia R., Perpar A., Metz M., Badakhshi H., Schreiber A., Nitsche M., Hipp P. (2016). Radiotherapy with 4 Gy × 5 versus 3 Gy × 10 for metastatic epidural spinal cord compression: Final results of the SCORE-2 trial (ARO 2009/01). J. Clin. Oncol..

[6] Rades D., Lange M., Veninga T., Stalpers L.J., Bajrovic A., Adamietz I.A., Rudat V., Schild S.E. (2011). Final results of a prospective study comparing the local control of short-course and long-course radiotherapy for metastatic spinal cord compression. Int. J. Radiat. Oncol. Biol. Phys..

[7] Barendsen G.W. (1982). Dose fractionation, dose rate and iso-effect relationships for normal tissue responses. Int. J. Radiat. Oncol. Biol. Phys..

[8] Joiner M.C., Van der Kogel A.J., Steel G.G. (1997). The linear-quadratic approach to fractionation and calculation of isoeffect relationships. Basic Clinical Radiobiology.

[9] Rades D., Panzner A., Rudat V., Karstens J.H., Schild S.E. (2011). Dose escalation of radiotherapy for metastatic spinal cord compression (MSCC) in patients with relatively favorable survival prognosis. Strahlenther. Onkol..

[10] Rades D., Hansen O., Jensen L.H., Dziggel L., Staackmann C., Doemer C., Cacicedo J., Conde-Moreno A.J., Segedin B., Ciervide-Jurio R. (2019). Radiotherapy for metastatic spinal cord compression with increased radiation doses (RAMSES-01): A prospective multicenter study. BMC Cancer.

[11] Rades D., Dunst J., Schild S.E. (2008). The first score predicting overall survival in patients with metastatic spinal cord compression. Cancer.

[12] Tomita T., Galicich J.H., Sundaresan N. (1983). Radiation therapy for spinal epidural metastases with complete block. Acta Radiol. Oncol..

[13] Baskin D.S., Evans R.W. (1996). Spinal cord injury. Neurology and Trauma.

[14] Chow E., Hoskin P., Mitera G., Zeng L., Lutz S., Roos D., Hahn C., van der Linden Y., Hartsell W., Kumar E. (2012). Update of the international consenus on palliative radiotherapy endpoints for future clinical trials in bone metastases. Int. J. Radiat. Oncol. Biol. Phys..

[15] Holland J.C., Andersen B., Breitbart W.S., Buchmann L.O., Compas B., Deshields T.L., Dudley M.M., Fleishman S., Fulcher C.D., Greenberg D.B. (2013). National Comprehensive Cancer Network. Distress management clinical practice guidelines in oncology. J. Natl. Comp. Cancer Netw..

[16] National Institutes of Health/National Cancer Institute (2010). Common Terminology Criteria for Adverse Events (CTCAE) Version 4.03.

[17] Franklin J.M., Eddings W., Austin P.C., Stuart E.A., Schneeweiss S. (2017). Comparing the performance of propensity score methods in healthcare database studies with rare outcomes. Stat. Med..

[18] Rades D., Cacicedo J., Lomidze D., Al-Salool A., Segedin B., Groselj B., Jankarashvili N., Conde-Moreno A.J., Schild S.E. (2022). A new and easy-to-use survival score for patients irradiated for metastatic epidural spinal cord compression. Pract. Radiat. Oncol..

[19] Marks L.B., Yorke E.D., Jackson A., Ten Haken R.K., Constine L.S., Eisbruch A., Bentzen S.M., Nam J., Deasy J.O. (2010). Use of normal tissue complication probability models in the clinic. Int. J. Radiat. Oncol. Biol. Phys..

[20] Emami B. (2013). Tolerance of the normal tissue to therapeutic irradiation. Rep. Radiother. Oncol..

[21] Steenland E., Leer J.W., van Houwelingen H., Post W.J., van den Hout W.B., Kievit J., de Haes H., Martijn H., Oei B., Vonk E. (1999). The effect of a single fraction compared to multiple fractions on painful bone metastases: A global analysis of the Dutch Bone Metastasis Study. Radiother. Oncol..

[22] Bone Pain Trial Working Party (1999). 8 Gy single fraction radiotherapy for the treatment of metastatic skeletal pain (randomised comparison with a multifraction schedule over 12 months of patient follow-up). Radiother. Oncol..

[23] Hartsell W.E., Scott C.B., Bruner D.W., Scarantino C.W., Ivker R.A., Roach M., Suh J.H., Demas W.F., Movsas B., Petersen I.A. (2005). Randomized trial of short- versus long-course radiotherapy for palliation of painful bone metastases. J. Natl. Cancer Inst..

[24] Roos D.E., Turner S.L., O’Brien P.C., Smith J.G., Spry N.A., Burmeister B.H., Hoskin P.J., Ball D.L., Trans-Tasman Radiation Oncology Group, TROG 96.05 (2005). Randomized trial of 8 Gy in 1 versus 20 Gy in 5 fractions of radiotherapy for neuropathic pain due to bone metastases (Trans-Tasman Radiation Oncology Group, TROG 96.05). Radiother. Oncol..

[25] Foro Arnalot P., Fontanals A.V., Galcerán J.C., Lynd F., Latiesas X.S., de Dios N.R., Castillejo A.R., Bassols M.L., Galán J.L., Conejo I.M. (2008). Randomized clinical trial with two palliative radiotherapy regimens in painful bone metastases: 30 Gy in 10 fractions compared with 8 Gy in single fraction. Radiother. Oncol..

[26] Guckenberger M., Mantel F., Gerszten P.C., Flickinger J.C., Sahgal A., Létourneau D., Grills I.S., Jawad M., Fahim D.K., Shin J.H. (2014). Safety and efficacy of stereotactic body radiotherapy as primary treatment for vertebral metastases: A multi-institutional analysis. Radiat. Oncol..

[27] Wong H.C.Y., Lee S.F., Chan A.W., Caini S., Hoskin P., Simone C.B., Johnstone P., van der Linden Y., van der Velden J.M., Martin E. (2023). Stereotactic body radiation therapy versus conventional external beam radiotherapy for spinal metastases: A systematic review and meta-analysis of randomized controlled trials. Radiother. Oncol..

[28] Guckenberger M., Dahele M., Ong W.L., Sahgal A. (2023). Stereotactic body radiation therapy for spinal metastases: Benefits and limitations. Semin. Radiat. Oncol..

[29] Hashimoto K., Nishimura S., Akagi M. (2021). Lung adenocarcinoma presenting as a soft tissue metastasis to the shoulder: A case report. Medicina.

[30] Bilsky M.H., Laufer I., Fourney D.R., Groff M., Schmidt M.H., Varga P.P., Vrionis F.D., Yamada Y., Gerszten P.C., Kuklo T.R. (2010). Reliability analysis of the epidural spinal cord compression scale. J. Neurosurg. Spine.

[31] Fisher C.G., DiPaola C.P., Ryken T.C., Bilsky M.H., Shaffrey C.I., Berven S.H., Harrop J.S., Fehlings M.G., Boriani S., Chou D. (2010). A novel classification system for spinal instability in neoplastic disease: An evidence-based approach and expert consensus from the Spine Oncology Study Group. Spine Phila Pa 1976.

[32] Fourney D.R., Frangou E.M., Ryken T.C., Dipaola C.P., Shaffrey C.I., Berven S.H., Bilsky M.H., Harrop J.S., Fehlings M.G., Boriani S. (2011). Spinal instability neoplastic score: An analysis of reliability and validity from the spine oncology study group. J. Clin. Oncol..

[33] Wänman J., Jernberg J., Gustafsson P., Abul-Kasim K., Grabowski P., Bobinski L., Crnalic S. (2021). Predictive Value of the Spinal Instability Neoplastic Score for Survival and Ambulatory Function After Surgery for Metastatic Spinal Cord Compression in 110 Patients with Prostate Cancer. Spine Phila Pa 1976.

[34] Laufer I., Iorgulescu J.B., Chapman T., Lis E., Shi W., Zhang Z., Cox B.W., Yamada Y., Bilsky M.H. (2013). Local disease control for spinal metastases following “separation surgery” and adjuvant hypofractionated or high-dose single-fraction stereotactic radiosurgery: Outcome analysis in 186 patients. J. Neurosurg. Spine.

[35] Turel M.K., Kerolus M.G., O’Toole J.E. (2017). Minimally invasive “separation surgery” plus adjuvant stereotactic radiotherapy in the management of spinal epidural metastases. J. Craniovertebr. Junction Spine.

[36] Ito K., Sugita S., Nakajima Y., Furuya T., Hiroaki O., Hayakawa S., Hozumi T., Saito M., Karasawa K. (2022). Phase 2 clinical trial of separation surgery followed by stereotactic body radiation therapy for metastatic epidural spinal cord compression. Int. J. Radiat. Oncol. Biol. Phys..

[37] Moussazadeh N., Laufer I., Yamada Y., Bilsky M.H. (2014). Separation surgery for spinal metastases: Effect of spinal radiosurgery on surgical treatment goals. Cancer Control.

[38] Versteeg A.L., van der Velden J.M., Hes J., Eppinga W., Kasperts N., Verkooijen H.M., Oner F.C., Seravalli E., Verlaan J.J. (2018). Stereotactic radiotherapy followed by surgical stabilization within 24 h for unstable spinal metastases; A stage I/IIa study according to the IDEAL Framework. Front. Oncol..

[39] Kirshblum S., Botticello A., Benedetto J., Donovan J., Marino R., Hsieh S., Wagaman N. (2020). A comparison of diagnostic stability of the ASIA Impairment Scale versus Frankel Classification Systems for traumatic spinal cord injury. Arch. Phys. Med. Rehabil..

